# McCune-Albright Syndrome: A Case From Mauritius

**DOI:** 10.7759/cureus.72810

**Published:** 2024-11-01

**Authors:** Vedprakash Seewoolall

**Affiliations:** 1 Internal Medicine, Sir Seewoosagur Ramgoolam National Hospital, Pamplemousses, MUS

**Keywords:** cafe au lait macules, denosumab, fibrous dysplasia, growth hormone excess, mccune-albright syndrome

## Abstract

McCune-Albright syndrome (MAS) is a rare genetic disease characterized by a triad of fibrous dysplasia, café-au-lait spots, and endocrine dysfunction. We present a 15-year-old male patient from Mauritius with MAS having multiple café-au-lait macules (CALM) and suffering from polyostotic fibrous dysplasia involving long bones of bilateral lower limbs and craniofacial deformities without endocrine abnormality. This case highlights the long-term management of MAS in a pediatric patient, focusing on both surgical interventions and the use of denosumab to address bone fragility.

## Introduction

McCune-Albright syndrome (MAS) is a rare, complex, non-hereditable genetic disease, which is defined by a wide range of clinical presentations, including bone involvement, café-au-lait macules (CALM), and endocrine abnormalities [1]. MAS has an estimated prevalence between 1/100,000 and 1/1,000,000 [2]. The non-classical form of the disease involves only two of the three above conditions. Gonadotropin-independent precocious puberty is the most common endocrine involvement described [3]. In this case report, we present a 15-year-old male presenting with CALM and fibrous dysplasia treated with denosumab. Data on the prevalence of MAS in Mauritius are limited, and understanding the associated treatment challenges would serve as a valuable reference for clinicians.

## Case presentation

**Figure 1:**
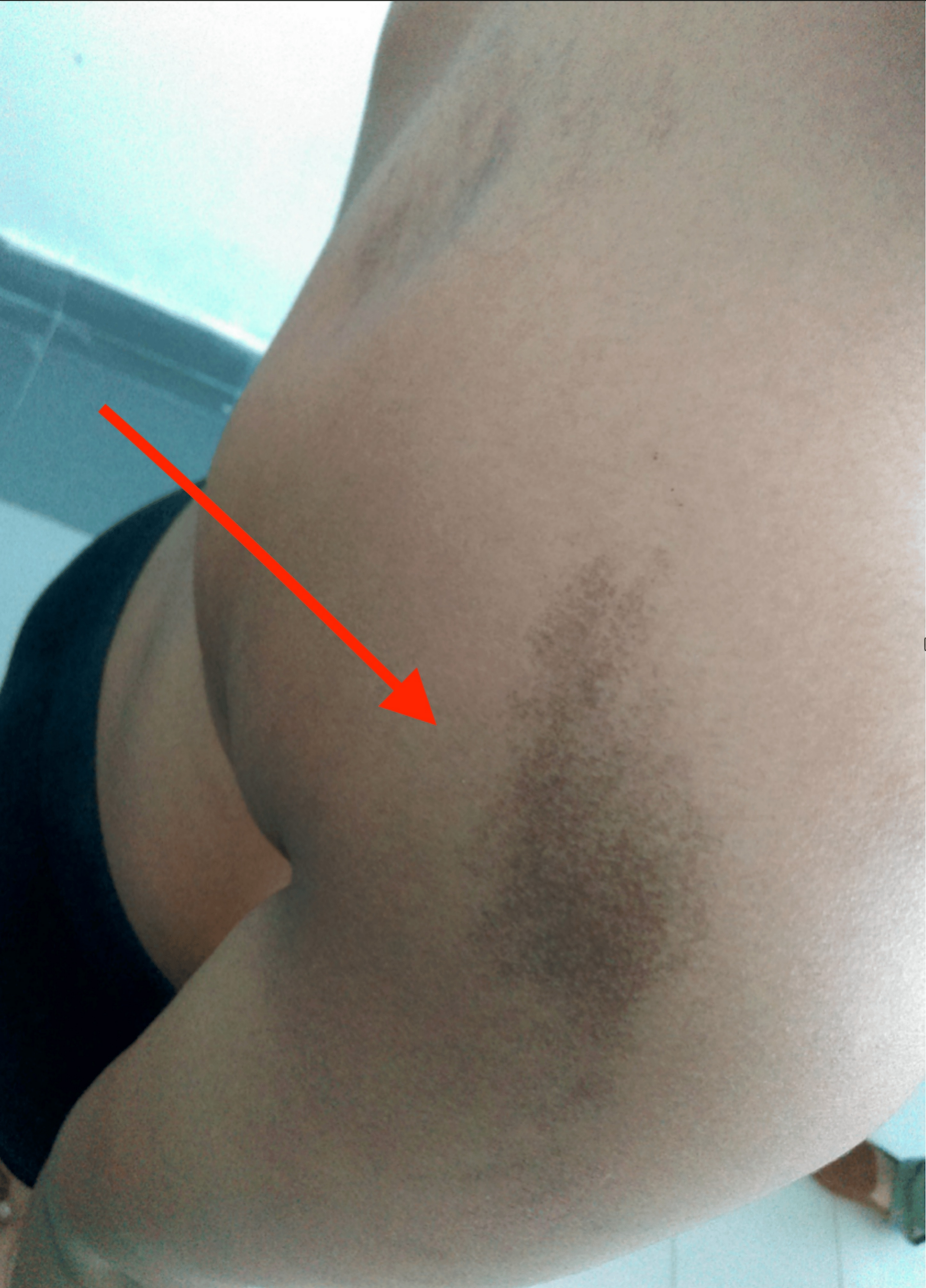
Café-au-lait macules

**Figure 2:**
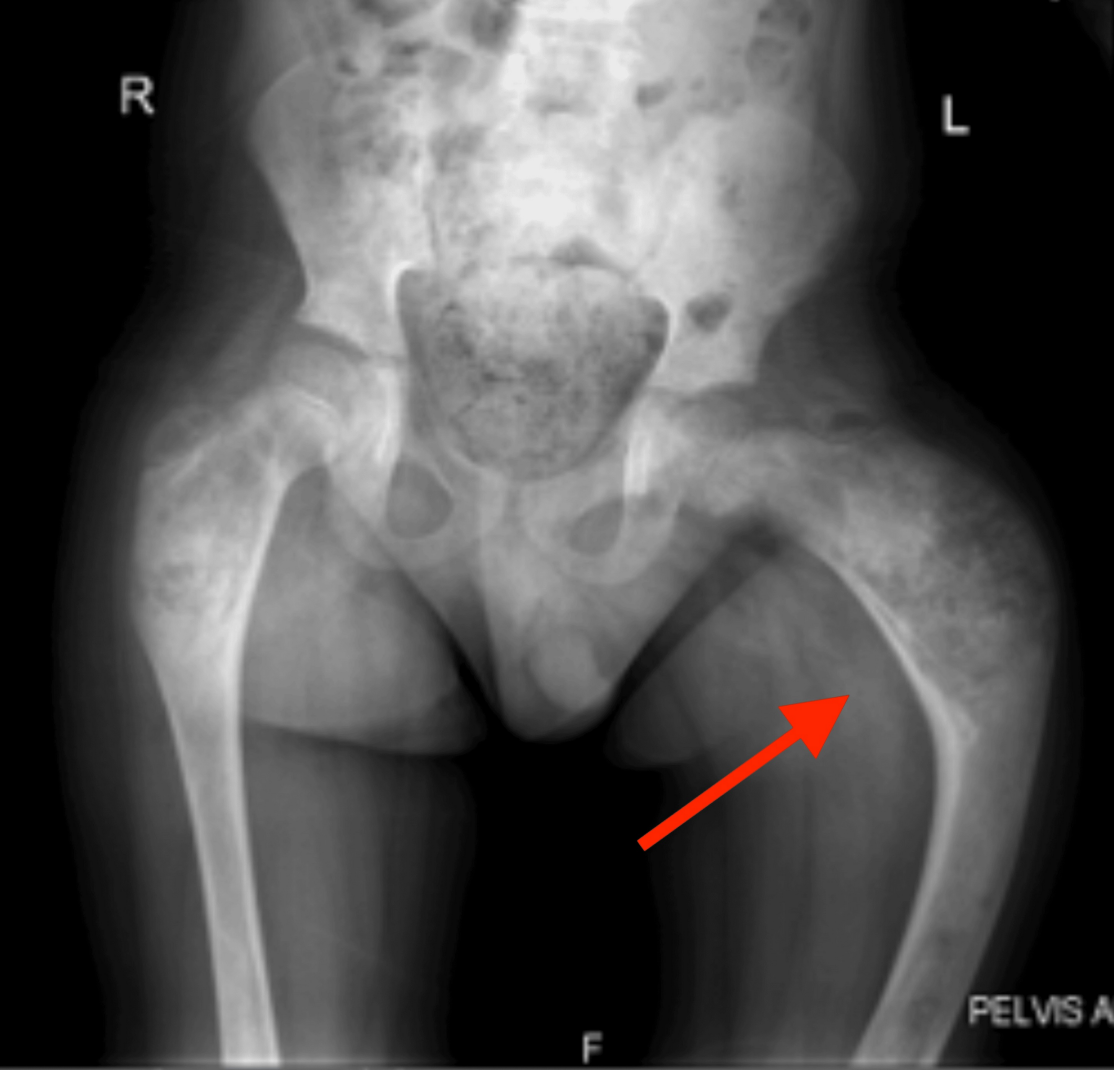
X-ray of the hip showing Shepherd's crook deformity

**Figure 3:**
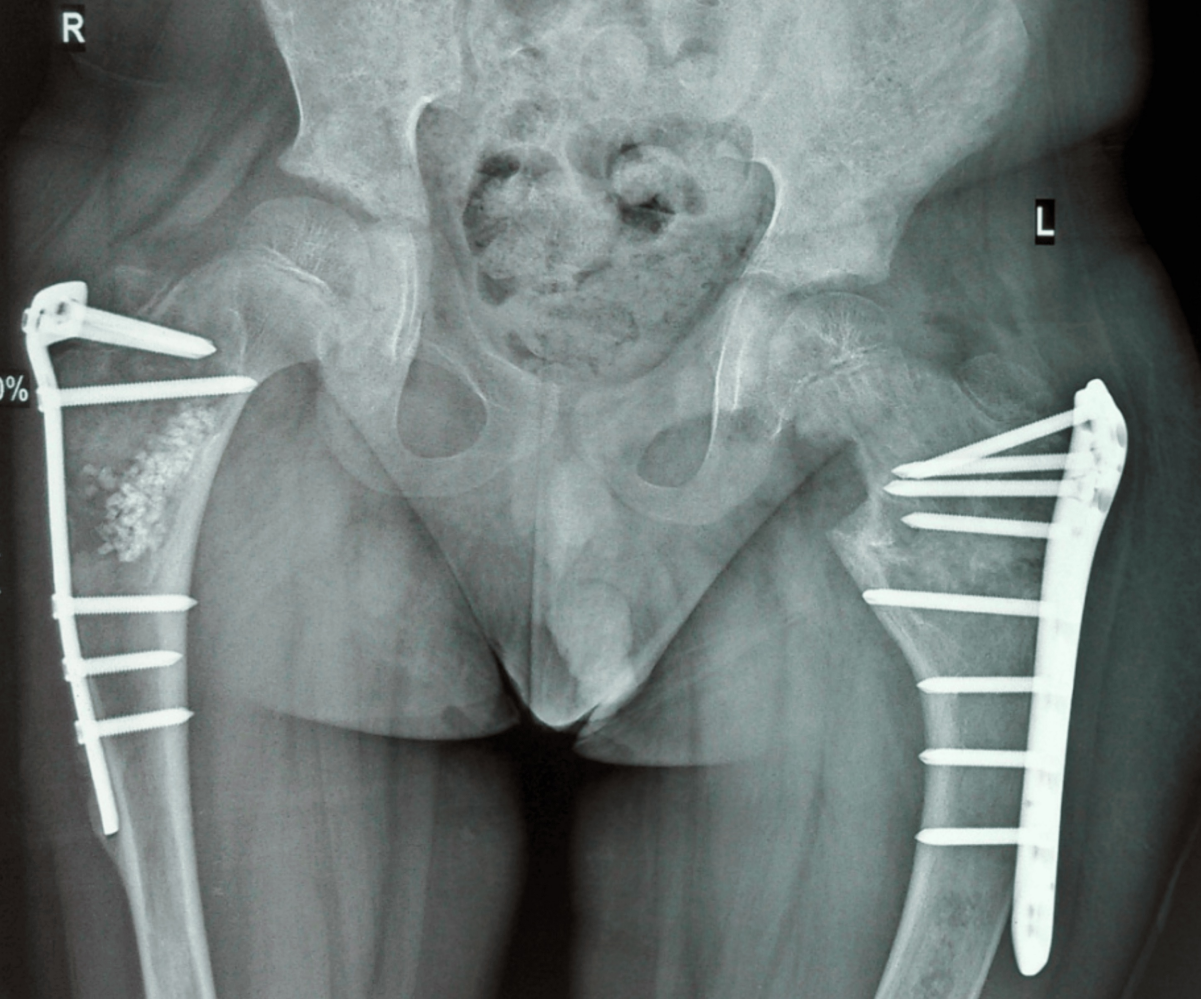
X-ray showing valgus osteotomy with open reduction and internal fixation and bone grafting

**Figure 4:**
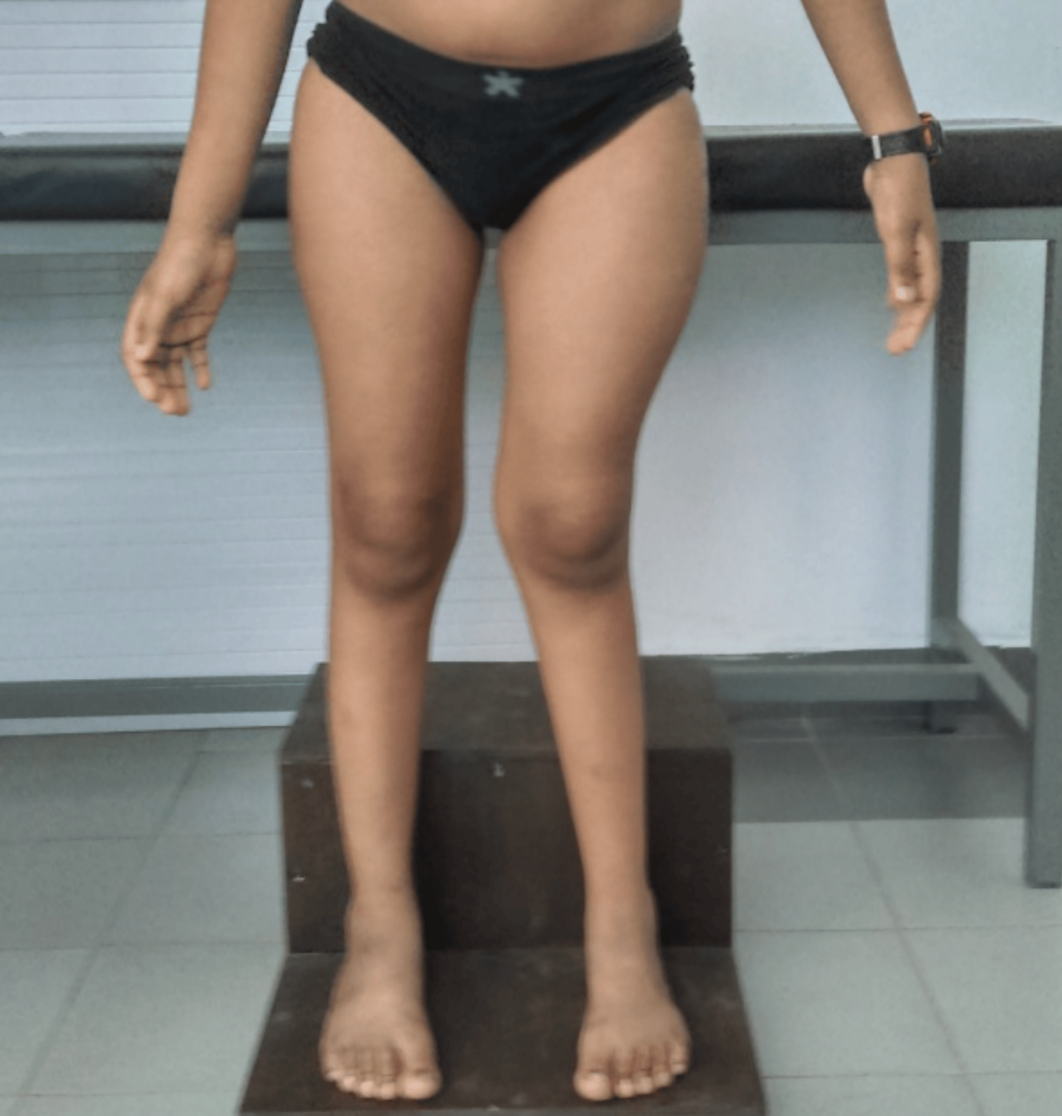
Patient aged 12 with progressive inward bowing of proximal lower limbs

**Figure 5:**
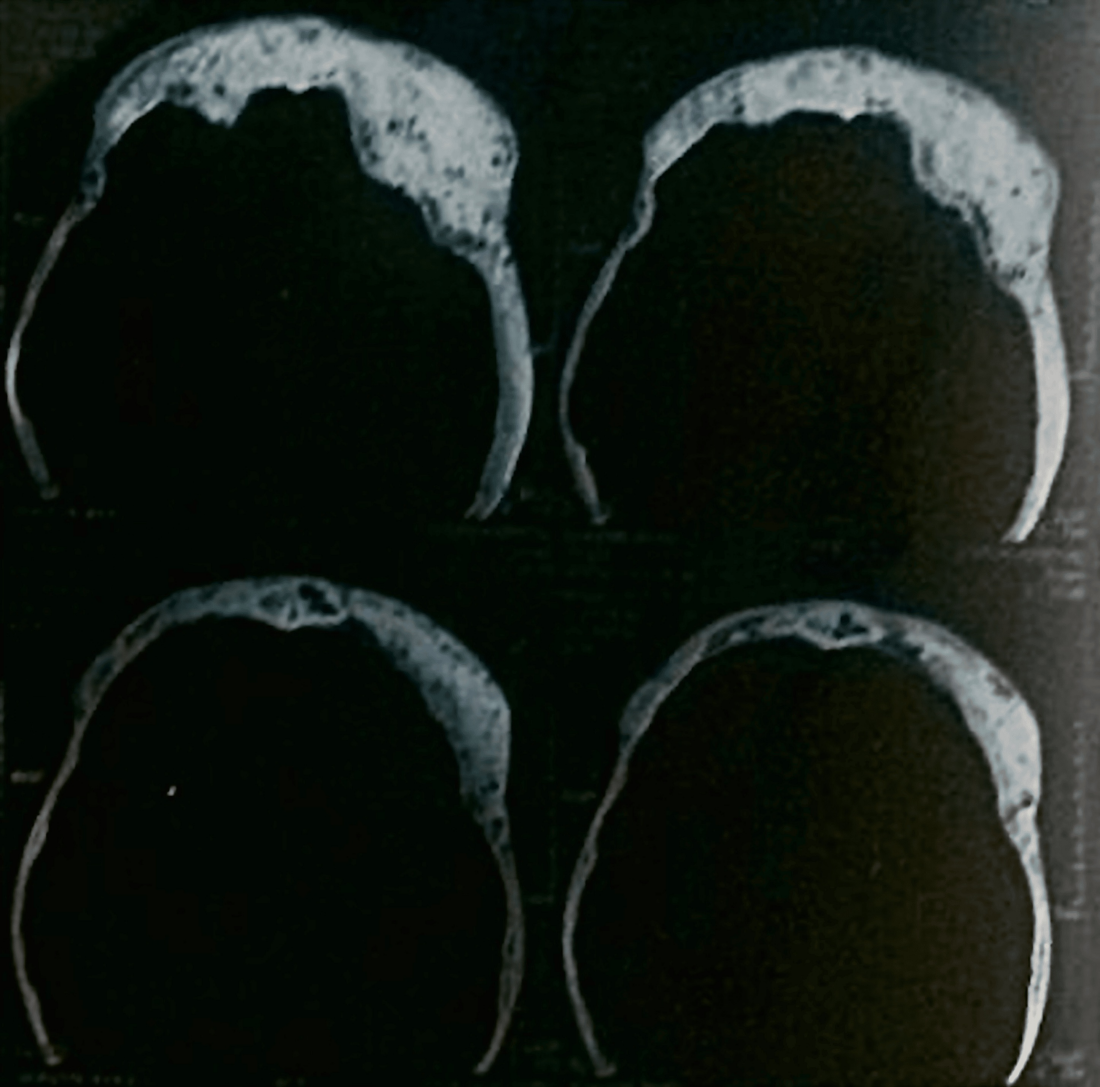
T2 MRI head post gadolinium showing cranial involvement of fibrous dysplasia

The patient with no history of consanguinity first presented at age three with deformities in both thighs, limping, and pain in the left thigh. Initial physical examination revealed CALM (Figure 1) on the right thigh, upper back, and arm, and X-rays showed expansile lesions in the proximal third of both femurs, indicative of Shepherd’s Crook deformity (Figure 2). At age seven, the patient traveled to India for further evaluation, where a biopsy of the left proximal femur confirmed fibrous dysplasia with 70% cartilaginous differentiation and 30% fibro-osseous components. He underwent bilateral proximal femur curettage, debridement, valgus osteotomy with open reduction and internal fixation, and bone grafting (Figure 3). The patient was regularly, followed by the orthopedic team in Mauritius, and underwent further corrective surgeries both in Mauritius and abroad. By age 12, he experienced chronic left hip pain and progressive proximal bowing of bilateral lower limbs (Figure 4). CT scans revealed altered bone density in the vertebrae, ribs, pelvis, and lower extremities, suggestive of polyostotic fibrous dysplasia. MRI of the brain revealed an involvement of the frontal, ethmoid, sphenoid, and left temporal bones, confirming craniofacial involvement (Figure 5). The pituitary and optic chiasma were normal. Hormonal tests showed no hyperfunctioning endocrine glands (FT4: 9.91 pmol/L, TSH: 1.35 mIU/L, cortisol AM: 345 nmol/L). The patient was recently started on denosumab, a receptor activator of NF-kappaB ligand (RANKL) inhibitor (currently on a loading dose of 60 mg once a month) to manage bone fragility and reduce fracture risk. He continues to be followed by a multidisciplinary team, including orthopedic surgeons, neurosurgeons, and endocrinologists. He will be regularly monitored to assess the benefits of denosumab on his bone health and growth.

## Discussion

Mutations in genomic alterations in the Gs alpha gene (GNAS), which encodes the α-subunit of the Gs G-coupled protein receptor cause a loss of the α-subunit's inherent GTPase activity. Constitutive receptor activation and inappropriate cAMP production arise from these mutations causing MAS [4]. Mosaicism accounts for the diverse clinical picture seen in patients with this syndrome [5].

The wide presentation and multisystem involvement of MAS pose distinct challenges in its management. CALMs are characterized by their unique localization as they are typically confined to one side of the body and do not cross the midline [3]. Interestingly, CALMs are also most prevalent on the side showing more bone disease.

The hallmark skeletal feature of MAS is fibrous dysplasia (FD), where normal bone is substituted and distorted by fibrous tissue. MAS most frequently affects the craniofacial bones, the femur, and bones of the pelvis [6]. The location of the bony involvement of FD is determined early; 90% of the total body skeletal disease burden is usually established by age 15 [7]. Polyostotic FD often leads to bone deformities, pathological fractures, and chronic pain. The most frequently affected bone in MAS is the proximal femur that develops a characteristic coxa vara deformity also called a "Shepherd's Crook" deformity [8]. In this patient, the progression of skeletal disease despite surgical interventions underscores the aggressive nature of MAS-related FD.

The presence of craniofacial involvement further complicates management, increasing the risk of functional and cosmetic issues. Imaging of the skull is paramount in the diagnosis of craniofacial FD. Indeed, a CT scan of the skull should initially be repeated each year and then less regularly once disease progression stabilizes [2].

Endocrine abnormalities are another cornerstone of MAS. While endocrine tests performed in this patient were normal, growth hormone hypersecretion is commonly seen in the second decade of life of MAS patients. Growth hormone excess usually responds well to somatostatin analogs such as octreotide and lanreotide or growth hormone receptor antagonists (pegvisomant), whether used individually or combined [9]. There is growing evidence that novel therapies may improve the severe clinical manifestation of MAS. Denosumab (RANKL inhibitor), initially used as a treatment for osteoporosis, has been added to the therapeutic medical regimen of MAS [10].

## Conclusions

This case emphasizes the importance of a multidisciplinary approach to MAS, involving regular monitoring, orthopedic surgical interventions, and endocrine management to optimize outcomes. The case of this 15-year-old male with MAS demonstrates the complexity of managing polyostotic FD, particularly in pediatric patients with craniofacial involvement. Surgical interventions remain crucial for correcting deformities. The use of denosumab, in this case, is notable as it represents a targeted approach to reduce bone resorption and improve bone density, addressing one of the key challenges in managing FD. Although denosumab has shown promise, its long-term efficacy and safety in pediatric patients with MAS need further evaluation.
